# Sex-specific outcomes and left atrial remodeling following catheter ablation of persistent atrial fibrillation: results from the DECAAF II trial

**DOI:** 10.1007/s10840-024-01831-w

**Published:** 2024-06-07

**Authors:** Hadi Younes, Christian Sohns, Nazem Akoum, Han Feng, Eli Tsakiris, Abdel Hadi El Hajjar, Eoin Donnellan, Amitabh C. Pandey, Chanho Lim, Ghassan Bidaoui, Mario Mekhael, Charbel Noujeim, Nour Chouman, Ala Assaf, Ghaith Shamaileh, Francisco Tirado Polo, Mayana Bsoul, Lilas Dagher, Omar Kreidieh, Swati Rao, Philipp Sommer, Mathias Forkmann, Johannes Brachmann, Nassir Marrouche, Christian Mahnkopf

**Affiliations:** 1 Tulane Research and Innovation for Arrhythmia Discoveries-TRIAD Center, Tulane University School of Medicine, New Orleans, LA USA; 2 Clinic for Electrophysiology, Herz- Und Diabeteszentrum NRW, Ruhr-Universität Bochum, Bad Oeynhausen, Germany; 3 Department of Cardiology, Klinikum Coburg, Coburg, Germany; 4 Medical School, University of Split, 21000 Split, Croatia; 5 Division of Cardiology, University of Washington, Seattle, WA USA

**Keywords:** Catheter ablation, Atrial fibrillation, Sex-specific differences, Quality of life

## Abstract

**Background:**

Catheter ablation is recognized as an effective treatment for atrial fibrillation (AF). Despite its effectiveness, significant sex-specific differences have been observed, which influence the outcomes of the procedure. This study explores these differences in a cohort of patients with persistent AF. We aim to assess sex differences in baseline characteristics, symptoms, quality of life, imaging findings, and response to catheter ablation in patients with persistent AF.

**Methods:**

This post hoc analysis of the DECAAF II trial evaluated 815 patients (161 females, 646 males). Between July 2016 and January 2020, participants were enrolled and randomly assigned to receive either personalized ablation targeting left atrial (LA) fibrosis using DE-MRI in conjunction with pulmonary vein isolation (PVI) or PVI alone. In this analysis, we aimed to compare female and male patients in the full cohort in terms of demographics, risk factors, medications, and outcomes such as AF recurrence, AF burden, LA volume reduction assessed by LGE-MRI before and 3 months after ablation, quality of life assessed by the SF-36 score, and safety outcomes. Statistical methods included *t*-tests, chi-square, and multivariable Cox regression.

**Results:**

Females were generally older with more comorbidities and experienced higher rates of arrhythmia recurrence post-ablation (53.3% vs. 40.2%, *p* < 0.01). Females also showed a higher AF burden (21% vs. 16%, *p* < 0.01) and a smaller reduction in left atrial volume indexed to body surface area post-ablation compared to male patients (8.36 (9.94) vs 11.35 (13.12), *p*-value 0.019). Quality of life scores were significantly worse in females both pre- and post-ablation (54 vs. 66 pre-ablation; 69 vs. 81 post-ablation, both *p* < 0.01), despite similar improvements across sexes. Safety outcomes and procedural parameters were similar between male and female patients.

**Conclusion:**

The study highlights significant differences in the outcomes of catheter ablation of persistent AF between sexes, with female patients showing worse quality of life, higher recurrence of AF and AF burden after ablation, and worse LA remodeling.

**Graphical Abstract:**

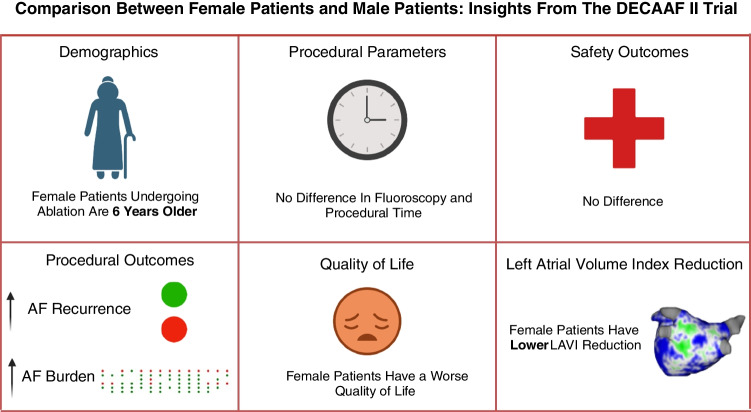

## Introduction

Catheter ablation is widely acknowledged as a safe and efficient therapeutic option for rhythm control of atrial fibrillation (AF). Sex-specific differences have been observed in the epidemiology, pathophysiology, presentation, and natural course of atrial fibrillation, similar to other cardiovascular conditions [1]. However, our understanding of the underlying pathophysiological basis for these differences and their implications for therapy and prognosis is still limited [2].

While catheter ablation is an effective option for restoring normal heart rhythm in many highly symptomatic patients, significant sex-specific differences have been reported [3]. When female patients undergo ablation, they are generally older, have longer AF episodes [1], failed multiple antiarrhythmic drugs, and have more comorbidities compared to male patients [3–6].

The impact of these differences on the outcomes of catheter ablation remains unclear, especially in persistent atrial fibrillation. In this DECAAF II post hoc analysis, we aimed to assess sex differences in baseline characteristics, symptoms and quality of life, advanced cardiac imaging findings, and response to catheter ablation in a cohort of persistent AF patients presenting for catheter ablation.

## Methods

### Main study design

The design and methodology of the DECAAF II trial have been previously published [7]. Between July 2016 and January 2020, patients with persistent AF were enrolled and randomly assigned to receive catheter ablation targeting left atrial (LA) fibrosis identified using Late-Gadolinium-Enhanced (LGE)–MRI in conjunction with pulmonary vein isolation (PVI), or PVI alone. All patients underwent an LGE-MRI within 30 days prior to ablation. LGE-MRIs were performed 3 months after the ablation to assess post-ablation LA changes including volumetric analysis, the extent of ablation-induced LA scar, and residual fibrosis [8].

### Subanalysis study design

This is a post hoc analysis of the main study where we sought to evaluate sex-specific differences in persistent atrial fibrillation (PeAF) presentation and catheter ablation (CA) outcomes. This subanalysis was approved by the institutional review board at Tulane University.

### Primary and secondary outcomes

The primary efficacy endpoint of the study was the first confirmed recurrence of any atrial tachyarrhythmia (aTA; including AF, flutter, or atrial tachycardia) lasting for > 30 s after a 90-day blanking period as previously described [9].

The primary safety composite outcome was defined by the occurrence of one or more of the following events during a 30-day post-ablation period: stroke, pulmonary vein stenosis, bleeding, heart failure, and death as described previously [10]. These safety endpoints were compiled from periprocedural complications reported by the operator within 30 days from ablation and were adjudicated by a 3-member outcomes committee based on the 2017 HRS consensus statement [11].

Secondary outcomes included quality of life as assessed by the SF-36 questionnaire, ablation and imaging parameters, and AF burden, calculated by dividing the total number of days in AF over the total number of days when strips were sent (secondary outcome) as previously described [12]. For our analysis, patients who transmitted under 30 ECG strips were excluded from AF burden calculations.

### Statistical analyses

Continuous and categorical variables were represented as mean (SD) and frequencies (percentage), respectively. Normality assumptions were checked among the continuous variables through the Sharpiro-Wilk tests, and then the Wilcoxon tests and *t*-tests were conducted accordingly to compare the group differences. A chi-square test was done to evaluate categorical variables. We performed a time-to-event analysis using the Kaplan–Meier to evaluate the primary outcome, and a multivariable analysis using Cox regression to assess predictors of AF recurrence.

## Results

### Demographics, risk factors, and medications

There were 815 patients included in this study with 161 female (21%) and 646 male (78%). The median follow-up duration for the overall cohort is 541 days (90 days blanking period + 451 days post blanking), with the interquartile range being 143.5–541 days. For males, the median follow-up duration is 541 (155–541) days, while for females being 365 (131–541) days. In terms of early dropout without an event occurring, there is no difference between males (23, 3.6%) and females (6, 3.6%). Female were older than male (66 [SD: 7] years vs 60 [SD: 9] years, *p* < 0.001), had more hypertension (72.2% vs 55.4%, *p* < 0.01), mitral valve disease (11.2% vs 4.6%, *p* < 0.01), but less coronary artery disease (7.7% vs 13.8%, *p* = 0.03), vascular diseases (4.7% vs 11.1%, *p* = 0.01), and tobacco use (27.2% vs 39.9%, *p* < 0.01), while being balanced in other risk factors (Table 1). Female were more likely to be prescribed digoxin (4.1% vs 1.4%, *p* = 0.02) and verapamil (3% vs 0.9%, *p* = 0.04) prior to the ablation. Furthermore, female patients were more likely to be prescribed antiarrhythmic drugs in the 30 days following the blanking period (22.5% vs 14.4%, *p* = 0.01).

**Table 1:** Baseline characteristics

	Female (*n* = 169)	Male (*N* = 646)	Total (*N* = 815)	*p*-value
Age (median)	66 (SD 7.4)	61 (SD 9.2)	62 (SD 9.1)	< 0.01
CHF	25 (14.8%)	133 (20.1%)	155 (19.0%)	0.12
HTN	122 (72.2%)	358 (55.4%)	480 (58.9%)	< 0.01
DM	18 (10.7%)	64 (9.9%)	82 (10.1%)	0.77
Stroke	18 (10.7%)	50 (7.7%)	68 (8.3%)	0.22
VascularDx	8 (4.7%)	72 (11.1%)	80 (9.8%)	< 0.05
Tobacco	46 (27.2%)	258 (39.9%)	304 (37.3%)	< 0.01
CAD	13 (7.7%)	89 (13.8%)	102 (12.5%)	< 0.05
CABG	1 (0.6%)	12 (1.9%)	13 (1.6%)	0.24
MitralValveDx	19 (11.2%)	30 (4.6%)	49 (6.0%)	< 0.01
Hyperlipidemia	57 (33.7%)	219 (33.9%)	276 (33.9%)	0.97
RheumaticFever	1 (0.6%)	9 (1.4%)	10 (1.2%)	0.40
Cardioverted	143 (84.6%)	541 (83.7%)	684 (83.9%)	0.78
LA volume (cc)	118 (SD 34)	135 (SD 42)	132 (SD 41)	< 0.01
LA volume index	62 (SD 18)	63 (SD 22)	63 (SD 21)	0.82

*CHF*, congestive heart failure; *HTN*, hypertension; *DM*, diabetes mellitus; *Dx*, diseases; *CAD*, coronary artery diseases; *CABG*, coronary artery bypass grafting; *LA*, left atrial

### Primary and secondary efficacy outcome

**Fig. 1:**
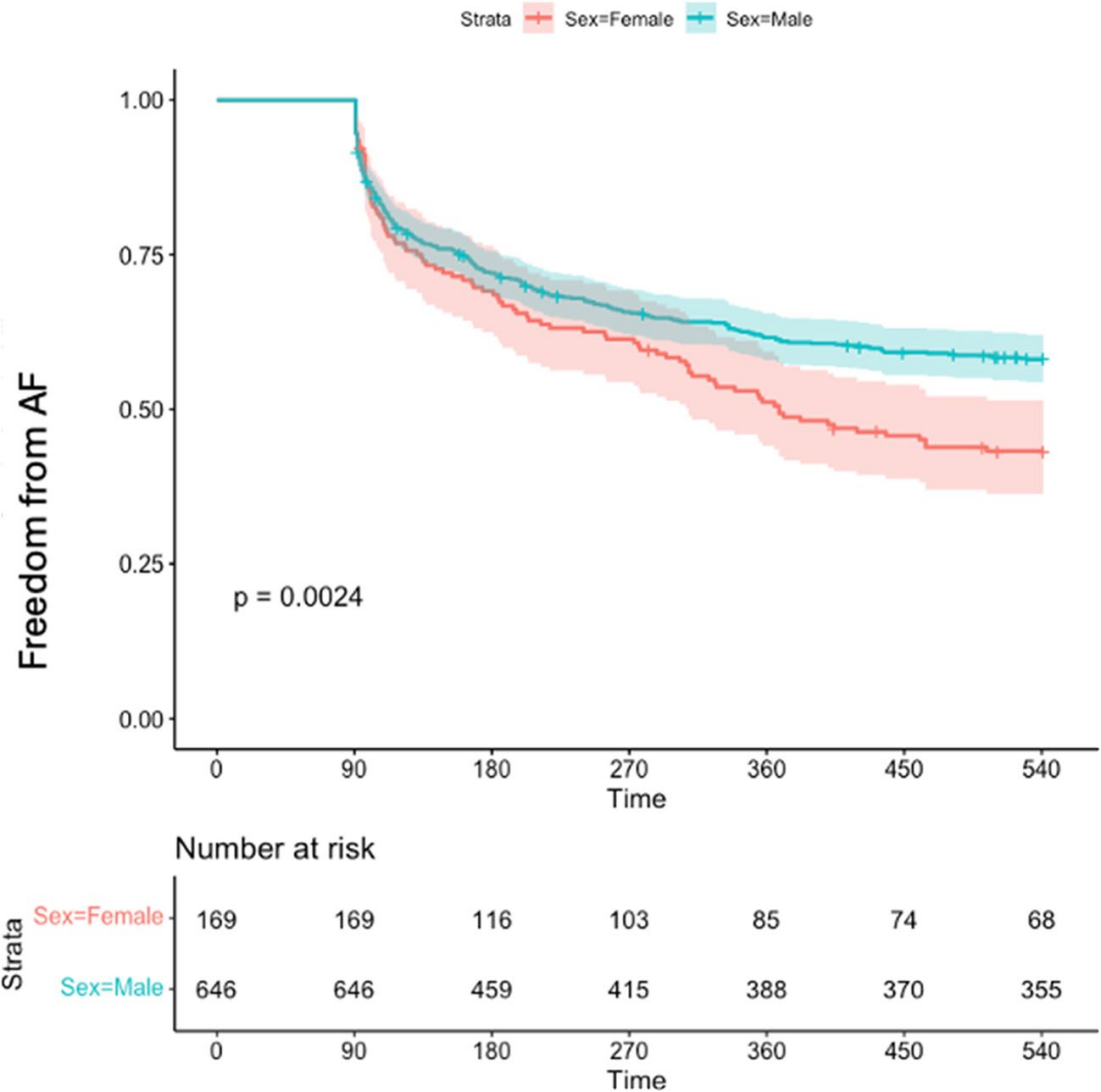
The Kaplan–Meier analysis for the primary outcome (female AF-free survival: 0.43 [0.36; 0.51]; male AF-free survival: 0.58 [0.54; 0.62]) [HR = 1.43 (1.13, 1.81)]

Females experienced more arrhythmia recurrence (53.3%) after the blanking period compared to males (40.2%) (*p* < 0.01), with a shorter average time to arrhythmia occurrence (260 [SD: 191] vs 296 [SD: 193] days) (*p* = 0.02). In time-to-event analysis, females had 43% more likelihood of experiencing the primary outcome of the study (Fig. 1) [HR = 1.43 (1.13, 1.81)].

**Fig. 2:**
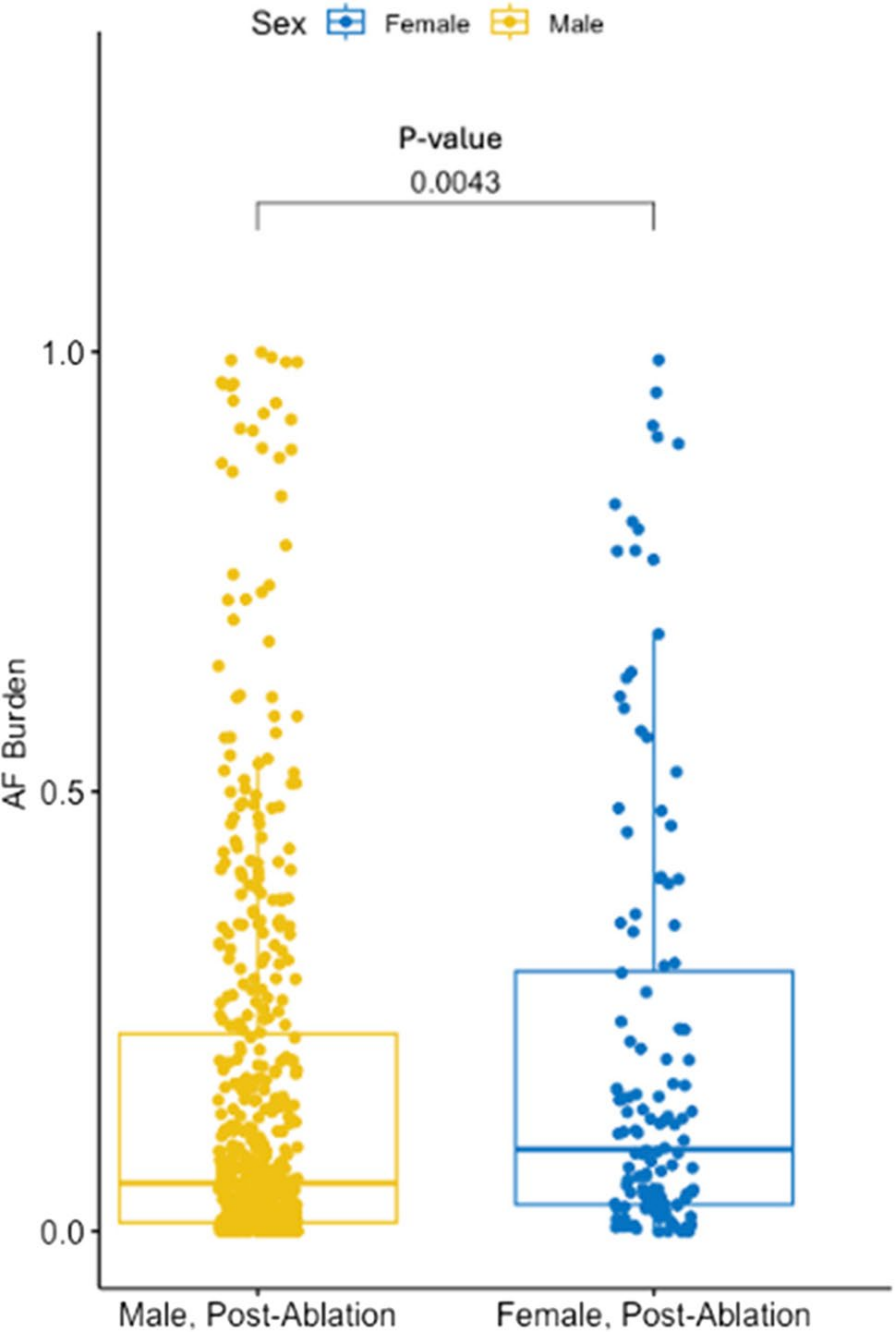
Scatter plot assessing AF burden after ablation in male and female

The mean total ECG submission among males is 396 (204), while for females, it is 402 (189). The *p*-value is 0.64. The mean total ECG submission after the blanking period among males and females is 314 (185) and 315 (166) respectively. The corresponding *p*-value is 0.76. We excluded 8 (4.7%) female and 21 (3.3%) male patients out of the study by setting the threshold of at least 30 strips. Following ablation, the median AF burden was higher in females (21% [SD 0.26] vs 16% [SD 0.23]) (*p* < 0.01) (Fig. 2). Multivariable Cox regression analysis demonstrated sex an independent predictor of AF recurrence following catheter ablation with a hazard ratio (HR) of 1.40 (*p* =  < 0.01, 95% CI: 1.09–1.80). Age was also demonstrated to be a significant independent predictor, with every 10 additional years increasing the risk of recurrence (HR = 1.18, *p* = 0.01, 95% CI: 1.04–1.34). Lastly, left atrial volume index was also an independent and significant predictor (HR = 1.09, *p* < 0.01, 95% CI: 1.05–1.13 for every 10 units). Other clinical factors, such as congestive heart failure, hypertension, diabetes mellitus, previous stroke, vascular disease, tobacco use, coronary artery disease, and hyperlipidemia, did not exhibit a significant predictive value in our model. The results of the multivariable analysis are detailed in Table 2.

**Table 2:** Multivariable analysis of the full cohort

	coef	se(coef)	Pr( >|z|)	Hazard ratio	Lower .95	Upper .95
Female	0.33	0.13	0.009	1.40	1.09	1.80
CHF	− 0.001	0.14	0.993	0.999	0.76	1.30
Age (per 10 years)	0.16	0.07	0.013	1.18	1.04	1.34
HTN	− 0.12	0.11	0.281	0.89	0.71	1.10
DM	− 0.16	0.19	0.381	0.85	0.59	1.22
Stroke	0.12	0.19	0.533	1.12	0.78	1.61
VascularDx	0.12	0.22	0.587	1.12	0.74	1.72
Tobacco	0.14	0.11	0.192	1.15	0.93	1.43
CAD	− 0.15	0.20	0.459	0.86	0.58	1.28
Hyperlipidemia	0.19	0.11	0.100	1.21	0.96	1.51
LA_Volume_Index (10 cc)	0.08	0.02	< 0.001	1.09	1.05	1.13

*CHF*, congestive heart failure; *HTN*, hypertension; *DM*, diabetes mellitus; *Dx*, diseases; *CAD*, coronary artery diseases; *CABG*, coronary artery bypass grafting; *LA*, left atrial

### Imaging and procedural parameters

#### Left atrial volume, fibrosis, and scar formation

Female had a smaller LA size (118 [SD: 34] vs 135 [SD: 41] *p* < 0.01). Baseline fibrosis was comparable between the two groups (19% [SD: 7%] vs 19% [SD: 7%], *p* = 0.86). Similarly, no significant differences were found between females and males in terms of ablation-induced scar (9.08% [SD: 5%]vs 9.78% [SD: 5%], *p* = 0.10) and residual fibrosis (14.89% [SD: 6%] vs 14.6% [SD: 6%], *p* = 0.57) (Table 3). Interestingly LA volume indexed to body surface area reduction at 3 months compared to baseline was lower in female compared to male (− 8.36 [9.94] vs. − 11.35 [13.12] *p*-value = 0.02).

**Table 3:** Imaging metrics

Aspect	Finding	Female	Male	*p*-value
Ejection fraction	Pre-ablation ejection fraction	56%	53%	** < **0.01
	Post-ablation ejection fraction	60%	58%	** < **0.01
	LVEFChange (post–pre)	3.82 (9.40)	4.93 (9.80)	0.91
Ablation parameters	Baseline impedance (Ohm)	126	145	** < **0.01
	Impedance drop (%)	5.05	6.03	** < **0.01
	Fluoroduration (min)	15	15	0.75
	Transseptal time (min)	129	133	0.46
	Sheath time (min)	157	164	0.19
	Repeat ablation (%)	17	16	0.63
MRI findings	LAVI reduction (LAVI at 3 months – baseline LAVI)	− 8.36 (9.94)	− 11.35 (13.12)	0.02
	Baseline fibrosis (%)	19	19	0.86
	Ablation scar (%)	9	10	0.10
	Residual fibrosis (%)	15	15	0.57

#### Procedural parameters

There were no significant differences in terms of fluoroscopy duration (15.30 min vs 15.08 min, *p* = 0.75), transseptal time (129.24 min vs 133.35 min, *p* = 0.46), and sheath time (157.23 min vs 164.05 min, *p* = 0.19) (Table 3). Regarding repeat ablation, we found no significant difference (17.2% vs 15.6%, *p* = 0.63) between the sexes in this cohort.

### Quality of life

**Fig. 3:**
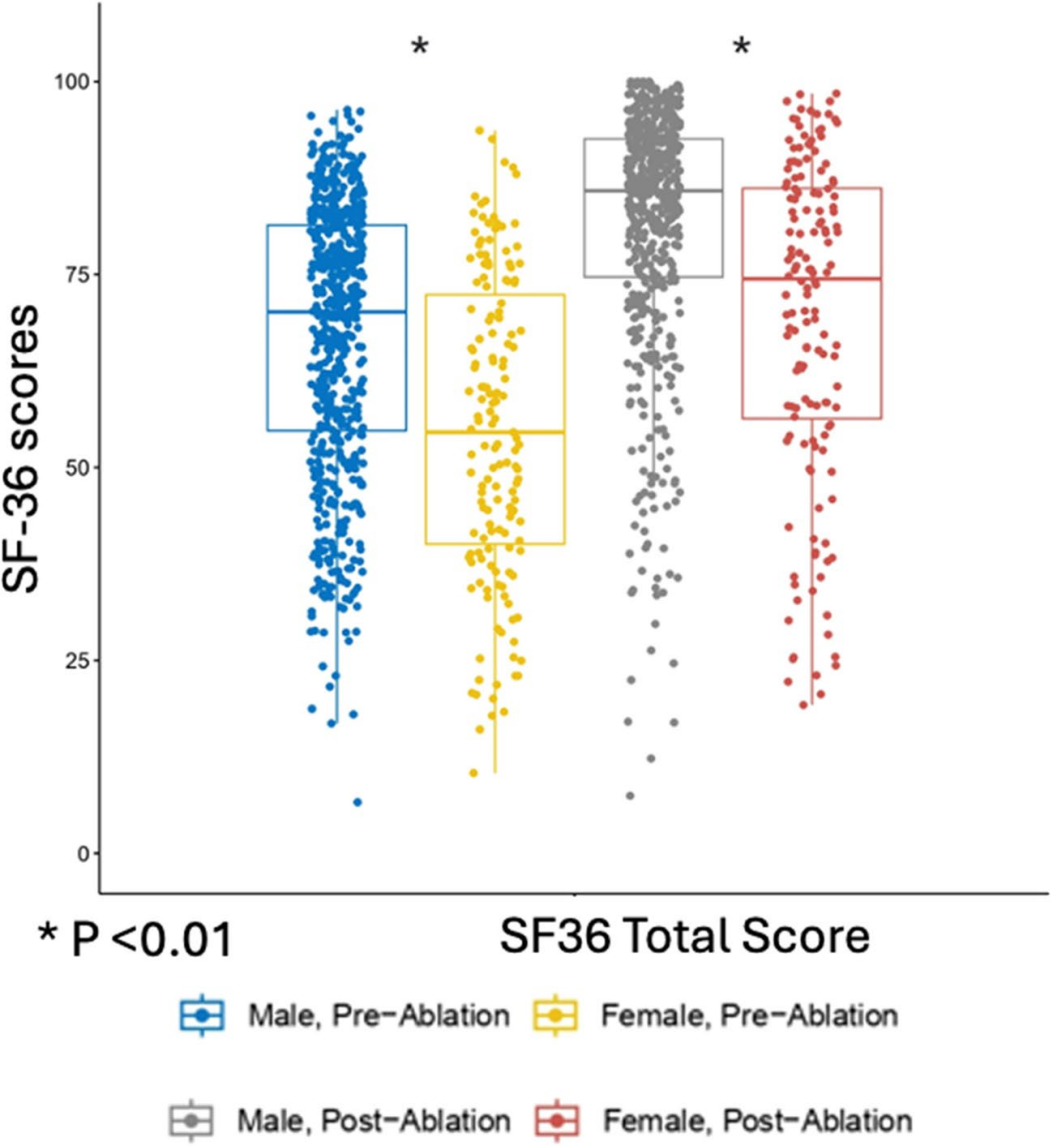
Quality of life assessment using the SF-36 pre- and post-ablation

Regarding quality of life, female patients reported significantly worse quality of life at baseline and the follow-up intervals, with no difference in improvement post-ablation. Pre-ablation, SF36 QoL scores were worse in female as the total score was lower (54 [SD: 20] vs 66[SD: 17]) (*p* < 0.01). Post-ablation at 12 months, SF36 QoL scores were also worse in female (69 [SD: 21] vs 81 [SD: 16]) (*p*-value < 0.01) (Fig. 3).

### Safety outcomes

There were no statistically significant differences in safety outcomes between the sexes. Death was reported in 2 male patients (0.31%) and 0 female patients (0%), stroke occurred in 5 male patients (0.8%) and 1 female patient (0.6%), while heart failure was reported by only 1 male patient, and no female patients. Esophageal injury was reported in 5 male patients and 1 female patient, and bleeding requiring transfusion was reported in only 1 male patient, with no cases reported in female patients. Tamponade was reported by 8 male patients and 2 female patients. No cases of pulmonary stenosis were reported in either male or female patients (Table 4).

**Table 4:** Safety outcomes were evaluated in the full safety population for the 30-day period following ablation. *p*-values were computed using the Fisher exact test

Safety outcomes	Female (*n* = 169)	Male (*n* = 646)	*p*-value
Bleeding requiring transfusion	0 (0.0%)	1 (0.2%)	0.61
Heart failure	0 (0.0%)	1 (0.2%)	0.61
Pulmonary vein stenosis	0 (0%)	0 (0%)	NA
Stroke/TIA	1 (0.6%)	5 (0.8%)	0.81
Death	0 (0.0%)	2 (0.31%)	0.46
Esophageal injury	1 (0.6%)	5 (0.8%)	0.81
Perforation/tamponade	2 (1.18%)	8 (1.24%)	0.95

*TIA*, transient ischemic attack

## Discussion

In this subanalysis of the DECAAF II clinical trial, we show [1] notable sex differences in age, quality of life measures, atrial volume, comorbidities, and post-ablation atrial changes; [2] female sex as an independent predictor of AF recurrence after ablation alongside LA volume and age; [3] female patients experience a smaller reduction in LA volume compared to male patients following ablation; and [4] we found no significant sex difference in safety outcomes.

The difference in quality of life may be due to females having higher average heart rates during AF and experiencing longer AF episodes compared to male [14]. Advanced treatments for symptomatic improvement are often not pursued in female patients with AF despite having relatively more severe symptoms [1].

Our study observed a notable age difference between female and male, with females being older by approximately 6 years (*p* < 0.001). This finding aligns with the FIRE and ICE trial post hoc analysis [15], where female participants were on average 4 years older than male (*p* < 0.01). The latter found that after catheter ablation of paroxysmal AF, female sex was associated with an almost 40% increase in the risks of atrial arrhythmia recurrence and cardiovascular rehospitalization. Females were more likely to be prescribed pharmacological therapy before ablation, potentially limiting or delaying their referral for ablation [13]. A subanalysis conducted from the CIRCA DOSE study, which also investigated sex-based differences in patients with paroxysmal AF, revealed no significant differences in AF recurrence rates. Both male and female patients exhibited similar freedom from any atrial tachyarrhythmia and symptomatic atrial tachyarrhythmia. Our subanalysis, however, included patients with persistent AF. This might account for the observed worse outcomes, given the generally higher AF burden in patients with persistent AF compared to those with paroxysmal AF. Despite these differences, certain findings were consistent across both studies. Notably, in both cohorts, female patients reported significantly lower symptom and quality of life scores compared to male patients, both at baseline and throughout the follow-up [14].

When analyzing LGE-MRI findings, there was no difference in the level of fibrosis, post-ablation scar, and residual fibrosis between the two sex groups. However, female patients showed less reduction in LA volume compared to males. We have previously shown that a less reduction in LA volume in the DECAAF II database is associated with more AF recurrence and AF burden, and worse quality of life [15]. This suggests a potentially less effective remodeling process after ablation in female patients that could contribute to the outcomes observed, raising the possibility of differing underlying substrates in female. In a study by Van Leuven et al. that included both persistent and paroxysmal AF patients, despite more extensive atrial remodeling, the AF recurrence rates were similar between male and female patients. However, low-voltage zones (LVZs) emerge as significant prognostic indicators in female patients only when the burden exceeds 15%. This indicates that there is more complexity to the LA substrate than previously understood, particularly in how it varies between female and male patients [16]. Further investigation is warranted to explore the various patterns of fibrosis, including patchy and interstitial fibrosis between female and male AF patients. For instance, previous work by Schotten et al. has identified a potential link between female sex and endomysial fibrosis, which was correlated with a worse underlying substrate [17].

In terms of ablation outcomes, multivariable analysis revealed age and LA volume as independent predictors of arrhythmia recurrence. The persistent identification of LA volume as an outcome predictor aligns with prior studies [18, 19].

The available data is insufficient to fully comprehend to the potential role of sex hormones in the progression of AF disease. Nevertheless, evidence suggests a link between low testosterone levels and an increased risk of AF, as well as the progression of atrial remodeling during AF in male patients [20]. Furthermore, the impact of hormone replacement therapy remains unclear. According to the Women’s Health Initiative (WHI), randomized postmenopausal female patients to either placebo or hormone therapy showed that hormone replacement did not significantly impact the incidence of AF [21]. Further research is essential to clarify the interplay between sex hormones and AF.

## Limitations

One of the limitations of this study is its post hoc nature, which resulted in a relatively smaller number of female compared to male as the initial enrollment was not balanced according to sex. Consequently, the findings may not be fully representative of the general population, as the study included more males and Caucasian patients than females and African American patients. Lastly, the reliance on patient electronic transmissions incurs a limitation within the AF burden metric.

## Conclusion

In conclusion, this post hoc analysis of the DECAAF II clinical trial has highlighted significant sex-specific differences in the outcomes of catheter ablation for persistent atrial fibrillation, with female patients typically being older when undergoing ablation, experiencing higher rates of arrhythmia recurrence, reporting worse quality of life measures both pre- and post-ablation, and exhibiting less reduction in left atrial volume post-ablation compared to male patients. Safety outcomes and procedural parameters were similar between male and female patients.

## Data Availability

The data supporting the findings of this study are sourced from the DECAAF II trial database, which was led by Dr. Marrouche, an author of this paper. The data are available upon reasonable request.
